# Bilateral Breast Metastases from Vulvar Carcinoma: A Case Report and Literature Review

**DOI:** 10.1155/2017/1357203

**Published:** 2017-04-12

**Authors:** D. C. Papatheodorou, C. G Liakou, K. Kalogerakos, Johannes Carl Athanasios Dimopoulos, N. Kalinoglou

**Affiliations:** ^1^Department of Gynecology, Metaxa Cancer Hospital, Piraeus, Greece; ^2^Breast Unit, Metaxa Cancer Hospital, Piraeus, Greece; ^3^Department of Radiotherapy, Metropolitan Hospital, Athens, Greece

## Abstract

Vulvar carcinoma is a rare disease that accounts for 3–5% of all gynecologic malignancies. Breast represents an unusual site of metastasis and only a few cases are reported. We describe the first case of bilateral metastatic breast carcinoma of vulvar origin, at an 80-year-old female patient. Six months after treatment of her primary disease, she presented with bilateral metastatic squamous cell breast carcinoma. Diagnosis was based on clinical, radiological, and histological facts. Breast although rare is another potential site of metastasis in vulvar cancer and thus mammary gland examination should be considered in the follow-up of these patients. Differential diagnosis between primary and metastatic lesions is of utmost importance for appropriate management.

## 1. Introduction

Vulvar cancer represents 3–5% of all gynecological malignancies and 1% of all cancers in females [1]. The disease is most commonly observed in postmenopausal women aged 65–70 years [2], presenting primarily with localized pruritus, vulvar mass, bleeding, or pain [3]. Squamous cell carcinoma is the predominant histological type, accounting for 90% [4], followed by melanoma, basal cell or Bartholin's gland carcinoma, sarcoma, and Paget's disease [5].

The dissemination pattern of vulvar neoplasms is mostly lymphogenic and the inguinal and femoral nodes are the primary sites of regional spread [6]. Direct extension to adjacent tissues may also occur, while hematogenous spread to distant organs presents late in the course of vulvar cancer and is rare in the absence of lymph node metastases [3].

Metastasis to the breast from vulvar carcinoma is extremely rare. To our knowledge, only two cases of unilateral breast metastasis have been reported [7, 8]. We report the first case of bilateral metastatic breast carcinoma of vulvar origin.

## 2. Case

An 80-year-old female patient presented in June 2015 with a 4.5 cm vulvar mass, involving the clitoris, the right labium majus, and the right lateral vaginal wall. Bilateral inguinal lymph nodes were palpable. The rest of the physical examination, including breast, was unremarkable. Her previous-year's mammogram was negative. Punch biopsy of the vulvar lesion was performed and the histological examination revealed a high grade, invasive vulvar squamous cell carcinoma. The preoperative evaluation included full blood count, biochemical profile, chest X-ray, and abdominal CT scan. There were no additional imaging findings and blood tests were within the normal range.

The patient underwent a radical vulvectomy and bilateral inguinal lymphadenectomy (triple incision technique). The histological report revealed an ulcerated tumor, measuring 4.5 × 4 × 3 cm, which showed features of poorly differentiated invasive squamous cell carcinoma. The surgical margins were free of disease (0,6 cm). Lymph node histopathology revealed tumor infiltration of a single ipsilateral lymph node, while all the lymph nodes dissected from the left groin showed no signs of malignancy. Vulvar lesion was, thus, classified stage IIIA (FIGO staging system) [2]. Patient's postoperative course was uncomplicated and she was discharged after twelve days of hospitalization. Postoperatively, the multidisciplinary team (MDT) suggested adjuvant radiation therapy and the patient received a total dose of 45 Gy to the groin and pelvis.

Follow-up was performed on an outpatient basis every three months. On her second visit, there were no signs of local recurrence. However, a palpable right breast mass at 9 o'clock position was noted. Mammography confirmed its presence and also revealed a second smaller mass in the contralateral breast. Fine-needle aspiration (FNAC) of both lumps was performed and cytological smears were stained with May-Grunwald-Giemsa and Papanicolaou. Microscopy revealed keratinized and nonkeratinized malignant squamous cells, with refractile cytoplasm, and individual cells lacking cohesion. The metastatic workup, including bone scan and abdominal, pelvic, and chest CT scan, was negative. Considering patient's medical history and the characteristic microscopic pattern of the lesions, diagnosis of metastatic squamous cell breast carcinoma, arising from the primary vulvar cancer, was established. Unfortunately, a few days later, she passed away at home from acute myocardial infarction.

## 3. Discussion

Vulvar cancer metastasizes through local spread, the lymphatic system, and the bloodstream [6]. The dissemination pattern is mostly lymphogenic. Lymphatic dye studies, by Parry-Jones [9], showed that dermal lymphatic network of the vulva courses to the superficial ipsilateral groin lymph nodes and then perforates the cribriform fascia to drain in the deep inguinal (femoral) nodes. Lymphatic drainage takes place from the lateral sites to the ipsilateral groin nodes, while drainage from the midline can be bilateral. At the level of the clitoris some lymph channels may drain directly to the pelvic lymph nodes. Sentinel lymph node (SLN) mapping investigates groin lymph node metastasis and permits less extensive node dissections and recognition of anatomic variations of the lymphatic channels [10, 11]. Distant metastases may occur either as a result of lymphatic embolization, in a stepwise fashion, to the pelvic and para-aortic lymph nodes or as a result of hematogenous spread, mainly to bones, liver, or lung [2]. They are uncommon in the initial presentation and encountered more frequently in the recurrent vulvar cancer, in 8% of patients [12].

The overall recurrence rate, according to Maggino et al., is 37.2% [12], while Gonzalez Bosquet et al. reported a rate of 26.7% [13]. The distribution of recurrences by site, in the former study, was 53.4% perineal, 18.7% inguinal, 5.7% pelvic, 7.9% distant, and 14.2% multiple. Tumor dimension, lymph node involvement, and stromal and lymph vascular space invasion were demonstrated as prognostic factors, predicting the risk of recurrence [12].

Breast carcinoma is the most common malignant tumor in females. However, metastatic tumors to the breast are rare and account for 0.5–1.5% of all breast cancers in clinical series and 6.6% in autopsy series [14, 15]. The commonest cause is spread from a contralateral breast carcinoma. Excluding hematologic malignancies, the most common primary sites are cutaneous melanoma, bronchogenic carcinoma, gastric carcinoma, and genitourinary tumors [16]. Among gynecologic cancers, the most frequent metastatic to the breast is ovarian cancer [17] but sporadic reports pertaining to other primary gynecologic malignancies, including cancers of the endometrium, cervix, fallopian tube, and choriocarcinoma, have been reported [15, 16, 18].

The breast is an exceptional site of metastasis from vulvar carcinoma. Only two cases have been described in the literature. In the first case, a 49-year-old woman, surgically treated for a stage II vulvar squamous cell carcinoma, presented 4 months after the operation with a 4 × 3 cm lump in the left breast [7]. The histological and immunohistochemical characteristics of the excisional biopsy specimen, the presence of human papilloma virus genome, demonstrated by in situ hybridization, in both vulvar and breast specimens and the absence of BRST2 confirmed the diagnosis. Surgical margins of the excisional biopsy were free and no further treatment was given. The patient remained free of disease for at least 73 months. In the second case, a 42-year-old patient, suffering from a stage IV_A_ vulvar squamous cell carcinoma, underwent radical vulvectomy and bilateral inguinal lymphadenectomy followed by external pelvic irradiation, as inguinal lymph nodes and the inferior surgical border were infiltrated by the tumor [8]. A recurrence in the left breast was confirmed by biopsy 3 months later. A modified radical mastectomy was performed and the patient received 3 cycles of adjuvant chemotherapy (cyclophosphamide, adriblastine, and cisplatin). She died at 11 months, in the context of cerebral metastasis.

The recognition of a breast lesion as primary or metastatic is crucial since it affects management and prognosis. A palpable, mobile, painless, well-circumscribed lump, localized at the upper outer quadrant, is the most common clinical presentation. Mammography usually depicts multiple or bilateral lesions, round and smooth, without speculation [15]. In contrast to primary breast carcinoma, the clinical and the radiological size of metastatic lesions coincide because of a minimal desmoplastic reaction around them [18]. Diagnosis is generally achieved histologically by fine-needle aspiration cytology or open biopsy [19]. FNA can differentiate between metastatic deposit and primary breast cancer, resulting in the avoidance of mastectomy and the implementation of the appropriate adjuvant therapy [20, 21].

In the current case, a typical aspect of squamous cell carcinoma associated with keratinization was observed in the breast lesions by FNA. Squamous cells are normally not found inside the breast. Thus, primary squamous cell carcinoma of the breast is a very rare tumor accounting for less than 0.1% of all invasive breast carcinomas and may arise from squamous metaplasia into an adenocarcinoma [22] or in cysts, abscesses, chronic inflammation, and fibroadenomas [23]. Its diagnosis requires the following: the absence of elements other than malignant epidermoid cells, the independence of the lesion from adjacent dermal structures, and the exclusion of other squamous carcinomas in the patient [8, 11, 24]. Consequently, our patient's medical history, in addition to the bilateral breast mass presentation, amplified the diagnosis of metastatic breast cancer.

The prognosis of breast metastasis is poor as widespread tumor dissemination is implied. Most patients die within the year following the diagnosis. Treatment is palliative, based on chemotherapy and sometimes comfort surgery [18].

## 4. Conclusion

This publication is an additional case of vulvar cancer metastasis to a rare location, the breast. Diagnosis was based on converging clinical, histological, and radiological facts. Differential diagnosis between primary and metastatic lesions is demanded for the appropriate and cost-effective patient management.
